# Impact of folic acid supplementation on ischemia‒reperfusion-induced kidney injury in rats: folic acid prophylactic role revisited

**DOI:** 10.1186/s12576-024-00900-z

**Published:** 2024-02-07

**Authors:** Aya E. H. Hamed, Sherif Khedr, Elsayed Ghonamy, Faten A. Mahmoud, Mona A. Ahmed

**Affiliations:** 1 Department of Medical Physiology, Faculty of Medicine, Ain Shams University, Cairo, Egypt; 2 Department of Histology and Cell Biology, Faculty of Medicine, Ain Shams University, Cairo, Egypt

**Keywords:** HMGB1, Inflammation, Acute kidney injury, Oxidative stress, Apoptosis

## Abstract

Folic acid (FA), with its anti-inflammatory and antioxidant properties, may offer protection against ischemia–reperfusion (IR) injury. This study investigated whether FA safeguards rat kidneys from IR by targeting high mobility group box-1 (HMGB1), a key inflammatory mediator. Fifty adult male Wistar rats were randomly allocated into four groups: control, IR, IR + FA pretreatment, and FA alone. Compared to controls, IR significantly impaired renal function and elevated levels of malondialdehyde, HMGB1, NF-κB, and caspase 3. FA pretreatment effectively reversed these detrimental changes, protecting renal function and minimizing tissue damage. The FA-alone group showed no significant differences compared to the control group, indicating no adverse effects of FA treatment. Mechanistically, FA inhibited HMGB1 expression and its downstream activation of NF-κB and caspase 3, thereby quelling inflammation and cell death. FA shields rat kidneys from IR-induced injury by suppressing HMGB1-mediated inflammation and apoptosis, suggesting a potential therapeutic avenue for IR-associated kidney damage.

**Supplementary Information:**

The online version contains supplementary material available at 10.1186/s12576-024-00900-z.

## Introduction

Acute kidney injury (AKI), defined as an abrupt loss of kidney function, is a serious life-threatening condition, especially among hospitalized patients, with a mortality rate of ∼ 50% caused by renal vascular, glomerular, and tubular damage [1]*.* Patients with AKI usually show complete recovery of their renal function; however, accumulating evidence suggests that AKI can subsequently trigger the onset of chronic kidney disease (CKD) in some patients and accelerate the progression of CKD in others. Renal ischemia‒reperfusion injury (RIRI) is a leading cause of AKI [2]*.* Unfortunately, it is not a rare situation; rather, it is encountered in multivarious clinical conditions, including renal transplantation, shock, sepsis, cardiac surgery, vascular surgery, and clamping of renal arteries during resection of renal tumors [3]*.* Ischemia followed by reperfusion fuels a series of biochemical and molecular changes that can elicit oxidative stress, inflammation, and apoptosis, paving the way for renal injury [4]*.* Mechanisms underlying RIRI are complex, intermingled, and usually act in a positive feedback fashion, causing permanent organ damage [5]*.*

One of the key players in postreperfusion renal injury is the generation of reactive oxygen species (ROS), as the imbalance between local oxygen supply and demand leads to their production. The formation of ROS leads to lipid peroxidation and oxidative damage to intracellular proteins and DNA [6]*.* The kidney is particularly vulnerable to oxidative stress since it occurs more intensely within it [7]*,* thus causing severe damage to tubular epithelial cells and eventually leading to necrotic and apoptotic cell death [5]. Therefore, embracing treatment protocols containing antioxidative agents to protect against IR-induced oxidative damage was very tempting [8]*.* Another mechanism through which ROS affect renal functionality is by triggering the inflammatory process [9]*.* In addition, renal IR per se fuels inflammation, causing more damage. The molecular mechanism behind this effect is very complicated; however, inhibiting the inflammatory response has also been a successful therapeutic tactic for preventing further injury.

Recently, the pathophysiological role played by HMGB1 in various diseases has drawn the attention of many researchers. It is an intranuclear molecule that can activate a sterile inflammatory process after extranuclear translocation. It is divided into three functional regions (A, B, and C). The B-box is a functional structural domain that causes an inflammatory response. This B-box has two crucial binding sites for Toll-like receptor 4 (TLR4) and receptor for advanced glycation end products (RAGE), which regulate the expression of proinflammatory cytokines. The biological activity of HMGB1 depends on its modifications, cellular location, redox state, and binding partners [10]*.* Previous literature documented a marked elevation in its level following IR [11]*,* which could boost a sterile inflammatory process. Therefore, it is plausible that lowering HMGB1 could ameliorate the deleterious impact of IR on the kidney.

FA, a member of the vitamin B complex, is essential for many biological functions and is required for optimal health, growth, and development. It acts as a cofactor in the one-carbon transfer reactions in the body and homocysteine (Hcy) remethylation pathway to reduce plasma Hcy levels and thus reduce Hcy-induced oxidative stress and DNA damage [12]. FA supplementation could reduce the risk of cardiovascular and hematological diseases, neurological and neuropsychiatric disorders, and several types of cancer [13]. Its favorable effects on health were reported to be related not only to the decrease in Hcy levels but also to other direct and indirect antioxidant activities [12]. In addition, it exhibits anti-inflammatory and anti-apoptotic properties as well as alleviation of endothelial dysfunction [13]*.* Supplementation with FA can increase cysteine and methionine levels and eventually glutathione (GSH) levels [14]*.* The direct antioxidant effect and the ability to inhibit lipid peroxidation indicate that FA is a multifunctional molecule that restores the antioxidant status of cells and tissues by normalization of GSH levels and direct scavenging of ROS [15]*.*

Unfortunately, there is no definitive successful treatment or preventive approach for RIRI, stemming from the lack of information about the exact underlying mechanisms. Mechanistic studies addressing the influence of FA in RIRI are scarce, and its multichannel protective role still needs to be fully explored. Our study aimed to delineate the potential protective effect of folic acid supplementation against IR-induced kidney dysfunction via affecting HMGB1 levels and the subsequent inflammatory process.

## Materials and methods

### Animals

Our study was conducted on 50 male adult (8 weeks old) Wistar albino rats initially weighing 180–220 g. Rats were purchased from the Vacsera Experimental Animal Facility (Helwan, Egypt). They were housed in the Faculty of Medicine Ain Shams University Research Institute (MASRI) animal house in plastic cages, with 4 rats per cage. Rats were left for 2 weeks before experimental procedures for acclimatization under standard conditions of boarding (room temperature 25 ± 2 °C, humidity 30–50%, and 12/12 h light/dark cycles). Rats were fed standard rat chow (prepared in our animal facility, composition shown in Additional file 1: Table S2 [16]) and were allowed ad libitum access to drinking water. The study was approved by the Research Ethical Committee of the Faculty of Medicine, Ain Shams University (FMASU 000017585).

The rats were randomly allocated into four groups: group I: sham-operated control group (*n* = 12). Rats in this group were left undisturbed in their cages for four weeks. At the end of the 4th week, rats were anesthetized using thiopental sodium (50 mg/kg) and then underwent identical surgical procedures as in the renal IR group but without clamping of the renal pedicles and were then studied 48 h after the sham operation.

Group II: folic acid-treated group (*n* = 12). Rats in this group received folic acid via oral gavage at a dose of 50 mg/kg once at 9 am for 6 days/week for 4 weeks. At the end of the 4th week, rats were anesthetized using thiopental sodium (50 mg/kg) and then underwent identical surgical procedures as the renal ischemia‒reperfusion group but without clamping of the renal pedicles and were then studied 48 h after the sham operation.

Group III: renal ischemia‒reperfusion group (*n* = 12). Rats in this group were left undisturbed in their cages for four weeks. At the end of the 4th week, rats were anesthetized using thiopental sodium (50 mg/kg) and then underwent warm renal ischemia for 45 min through bilateral clamping of the renal pedicles followed by 48 h of reperfusion.

Group IV: renal ischemia‒reperfusion treated with folic acid group (*n* = 14). Rats in this group received folic acid for four weeks as in the FA group. At the end of the 4th week, rats were anesthetized using thiopental sodium (50 mg/kg) then underwent renal IR as in the ischemia‒reperfusion group, followed by 48 h of reperfusion.

At the end of the experiments, rats were euthanized by opening the chest cavity under anesthesia.

### Folic acid treatment

Folic acid was obtained as tablets, each containing 5 mg supplied by El-Nile Co. for Pharmaceuticals and Chemical Industries, Egypt. Tablets were ground and dissolved in distilled water to form a yellowish solution with a concentration of 5 mg/ml water. FA was administered to the assigned groups by oral gavage at a dose of 50 mg/kg/day [17] at 9 am, for 6 days/week for 4 weeks [18].

### Estimation of kidney function in the serum

Creatinine levels were measured in serum by a quantitative colorimetric method using a QuantiChrom Creatinine Assay Kit (DICT-500), supplied by BioAssay Systems, Hayward, CA, USA, following the manufacturer’s instructions and according to the modified Jaffe method [18]*.*

Urea levels were measured in serum by a quantitative colorimetric method according to [20] using a QuantiChrom Urea Assay Kit (DIUR-500), supplied by BioAssay Systems, Hayward, CA, USA, according to the manufacturer’s instructions.

### Preparation of renal tissue homogenate

On the day of preparation, the left kidney was allowed to thaw, blotted with filter paper, and then weighed. The kidney was homogenized in 10 ml cold buffer (50 mM potassium phosphate, pH 7.5, 1 mM EDTA) per gram tissue, using MICCRA GmbH® homogenizer (Heitersheim, Germany). The sample was centrifuged at 5000 rpm for 15 min at 4 °C. Then, the supernatant was removed and stored at − 80 °C for later determination of malondialdehyde, superoxide dismutase, nuclear factor-kappa B, high mobility group box 1, caspase-3, and mitochondrial membrane potential. We followed the manufacturer’s instructions in each test with the recommended amount of the whole wet tissue, compared to the reagents amount and concentration provided with the kit.

### Determination of plasma total homocysteine level

Quantitative determination of total Hcy levels in plasma was performed by an enzyme immunoassay (ELISA) technique using CUSABIO Technology (CSB-E13376r) LLC, Houston, TX 77054, USA, following the manufacturer’s instructions. This assay employs the quantitative sandwich enzyme immunoassay technique [19]*.*

### Measurement of oxidative stress in renal tissue

Malondialdehyde (MDA) was measured in kidney tissue by a colorimetric method according to [22] using kits (MD 25 28) supplied by Biodiagnostics, Egypt.

Superoxide dismutase (SOD) enzyme activity in renal tissue was measured by a colorimetric method according to [20] using kits (SD 25 20) supplied by Biodiagnostics, Egypt. This assay relies on the ability of SOD to inhibit the phenazine methosulphate-mediated reduction of the microblue tetrazolium dye.

### Determination of the renal apoptotic marker and cell viability

Quantitative determination of caspase-3 levels (apoptosis indicator) in renal tissue was performed by enzyme immunoassay (ELISA) according to the manufacturer’s instructions using a rat active caspase-3 (A-CASP3) ELISA kit (MBS7244630) supplied by MyBioSource, San Diego, CA, USA.

Mitochondrial membrane potential (MMP), which is a cellular viability indicator, was studied by a fluorescence-based assay using a mitochondrial membrane potential assay kit (E-CK-A301) with JC-1, supplied by Elabscience Biotechnology Inc., Houston, Texas, USA, according to the manufacturer’s instructions.

### Measurement of HMGB1 levels and inflammatory markers in renal tissue

Quantitative determination of high mobility group box 1 (HMGB1) levels in kidney tissue was performed by an enzyme immunoassay (ELISA) technique according to the manufacturer’s instructions using a rat HMGB1 protein ELISA kit (MBS729203), supplied by MyBioSource, San Diego, CA, USA.

Quantitative determination of nuclear factor kappa-B (NF-κB) levels in kidney tissue was performed by an enzyme immunoassay (ELISA) technique according to the manufacturer’s instructions using a rat NF-κB ELISA Kit (MBS015549), supplied by MyBioSource, San Diego, CA, USA.

### Histopathological analysis

Hematoxylin and eosin staining (H&E), mac Manus Periodic acid Schiff’s (PAS) reaction, and modified Masson's Trichrome Technique were employed. Ten sections were examined of each group using each staining and compared to each other by the subjective findings under the microscope (Zeiss®) and further measurements using Image Analyzer program linked to Olympus® microscope to compare specific findings in the Histology and Cell Biology Department. The kidney histopathological changes were quantified using the Endothelial, Glomerular, Tubular, and Interstitial (EGTI) scoring system, described for animal research on injured renal tissue which evaluates the injury level in these 4 parameters [21]

### Statistical analysis

All analyses are expressed as the means ± SEM and analyzed using the statistical package for GraphPad Prism version 9.2.0 for Windows (GraphPad Software, San Diego, California, USA). Significance tests were two-tailed, and one-way ANOVA followed by Tukey’s test was performed. Correlation between the results of each test and the other regardless of the group (pooled data correlation) was performed following Pearson’s correlation and simple linear regression tests. In all statistical comparisons, a *p* value < 0.05 was used to indicate a statistically significant difference.

## Results

### Changes in renal function parameters and indicators of kidney injury

**Fig. 1:**
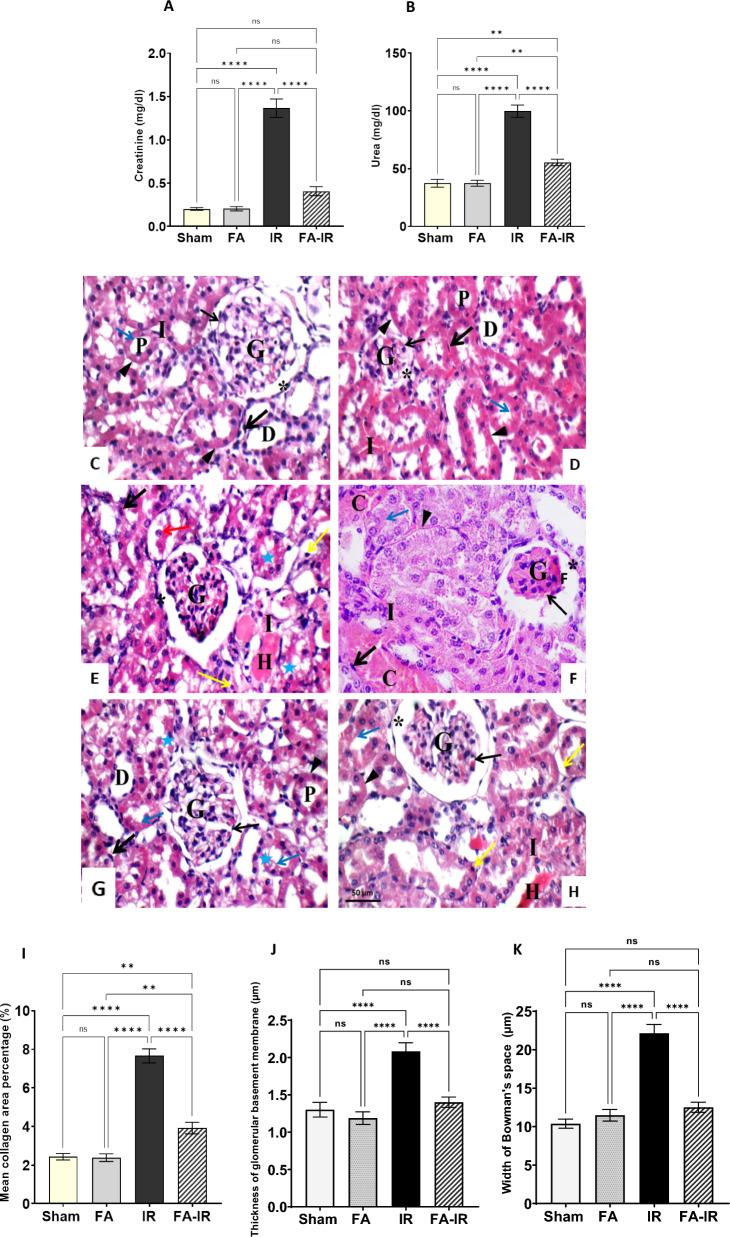
Biochemical and histological assessment of renal function and structure in the different studied groups. **A**, **B** Serum creatinine (**A**) and serum urea (**B**) levels. **C**–**H** H&E staining for the sham group (**C**) and FA group (**D**) showing the renal corpuscle, closely packed renal proximal tubules, and distal convoluted tubule. The glomerulus appeared normal and was surrounded by the thin-walled parietal layer of the Bowman capsule with no retraction of the tuft. The brush border of the tubules is seen intact (blue arrow) with a thin basement membrane. IR group (**E**, **F**) showing the glomerulus surrounded by the thickened parietal layer of Bowman’s capsule and retraction of the glomerular tuft. Most of the tubules are disrupted, showing a thickened basement membrane with loss of brush border (blue arrow) in more than 25% of the tubules with shedding of tubular cells (red arrow) and hyaline cast formation. Vacuolations are also seen in many tubules (blue star) with small dark pyknotic nuclei (yellow arrow). The interstitium is widened by necrotic cells and congested capillaries in up to 60% of the tissues. The endothelial cells are swollen (thick black arrow). FA-IR group (**G**, **H**) showing the glomerulus with a thin parietal layer of Bowman’s capsule and no retraction in the glomerular tuft. The proximal and distal tubules are closely packed in most of the fields, showing preserved brush borders (blue arrow) and thin basement membranes. There is loss of brush border in less than 25% of the tubules. The interstitial space appears with hemorrhage in less than 25% of the tissues. Some tubules appeared with cytoplasmic vacuolations (blue star) and small dark pyknotic nuclei (yellow arrow). The endothelium is intact with flat nuclei (thick black arrow). **I**–**K** Graphical summary for some features of kidney injury, mean of area percentage filled with collagen (**I**), thickness of basement membrane (**J**), width of Bowman’s space (**K**). Values are expressed as the mean ± SEM. ns: nonsignificant, **p < 0.01, ****p < 0.0001, [P] proximal tubules, [D] distal convoluted tubule, [G] glomerulus, [*] Bowman’s capsule, [↑] capillary tuft, [▲] basement membrane, [C] capillaries, [I] renal interstitium. Scale bar is 50 µm

In Fig. 1A, the serum creatinine level showed a statistically significant increase in the renal IR group compared to both the sham-operated group (sham) and folic acid-treated group (FA) (*p* < 0.0001 for both). In the folic acid-treated renal ischemia‒reperfusion group (FA-IR), serum creatinine levels were significantly decreased compared to those in the IR group (*p* < 0.0001) but were not significantly different from those in the sham or FA group. Meanwhile, the serum creatinine level in the FA group was comparable to that in the sham group.

Parallel to the changes observed in serum creatinine, serum urea levels were increased in the IR group compared to both control groups (*p* < 0.0001 for both). Interestingly, the FA-IR group showed a significant decrease in serum urea compared to the IR group (*p* < 0.0001). Meanwhile, it remained significantly higher than the serum urea level in both the sham and FA groups (*p* < 0.01 for both). Again, FA treatment did not change the serum urea level compared to that in the sham group (Fig. 1B).

Histological analysis confirmed our biochemical findings. H & E staining in both control groups showed a normal glomerular appearance surrounded by a thin-walled parietal layer of the Bowman capsule with a normal tuft of capillaries, an intact brush border of the tubules, and a thin basement membrane. There were no signs of interstitial inflammation or necrosis, and the endothelium of peritubular capillaries was uniform with flat nuclei and no swelling or disruption (Fig. 1C, D). Moreover, Masson trichrome staining showed minimal collagen deposition in the renal interstitium (Additional file 1: Fig. S1A, B), and PAS staining showed intact proximal convoluted tubule (PCT) brush borders and normal basement membranes of the renal tubules and parietal layer of the Bowman capsule (Additional file 1: Fig. S2A, B). In contrast, the IR group showed glomeruli surrounded by a thickened parietal layer of the Bowman capsule and retraction of the glomerular tuft, disruption in most of the tubules with thickened basement membrane. Loss of brush border in more than 25% of the tubules with shedding of tubular cells and hyaline cast formation was observed. Vacuolations are also seen in many tubules with small dark pyknotic nuclei and necrotic cells. Additionally, swollen endothelial cells were observed (Fig. 1E, F). Meanwhile, there was an increase in collagen fiber deposition around the renal corpuscle, in between renal tubules, and in the lumen of some tubules. Furthermore, there was a loss of the brush border of the cells of PCTs and an increased PAS reaction in the basement membrane of the tubules and the parietal layer of the Bowman capsule (Additional file 1: Fig. S1C, 2C). Interestingly, FA treatment in the IR group ameliorated and hindered all the histological deteriorations documented in the IR only group (Fig. 1G, H, Additional file 1: Fig. S1D, 2D). Quantitative analysis of the histological images showed a significant elevation in collagen deposition, thickening of the basement membrane, and width of Bowman’s space in the IR group compared to all other groups. Except for collagen deposition, FA treatment with IR could normalize the other parameters, rendering them nonsignificant compared to the control group; however, it could reduce collagen deposition significantly compared to the IR group (Fig. 1I–K). Table 1 and Additional file 1: Table S1 present the kidney injury score, which again underscores the significant renoprotective influence of FA against IR-induced injury.

**Table 1:** Cumulative values of kidney histopathological scores in the different studied groups

Group	Glomerular	Tubular	Tubule/interstitial	Endothelial
	Score			
Group I (Sham treated)	0.00 ± 0.00 *n* (10)	0.00 ± 0.00 *n* (10)	0.00 ± 0.00 *n* (10)	0.00 ± 0.00 *n* (10)
Group II (Folic acid)	0.00 ± 0.00 (10)	0.00 ± 0.00 (10)	0.00 ± 0.00 (10)	0.00 ± 0.00 (10)
Group III (Ischemia-reperfusion)	1.9 ± 0.17 *n* (10) a* b* d*	2.6 ± 0.22 *n* (10) a* b* d*	2.5 ± 0.22 *n* (10) a* b* d*	1.2 ± 0.13 *n* (10) a* b* d*
Group IV (Folic acid-ischemia reperfusion)	0.40 ± 0.22 *n* (10) a^ns^ b^ns^ c*	1.3 ± 0.21 *n* (10) a* b*c*	1.1 ± 0.10 *n* (10) a* b*c*	0.30 ± 0.21 *n* (10) a^ns^ b^ns^ c*

Data are presented as the mean ± standard error of the mean (SEM), *n* = number of sections examined/group. Statistical significance is expressed as ns: nonsignificant, *p < 0.05. (a: vs. Group I, b: vs. Group II, c: vs. Group III, and d: vs Group 4)

### Changes in plasma total homocysteine and oxidative stress parameters in renal tissue

**Fig. 2:**
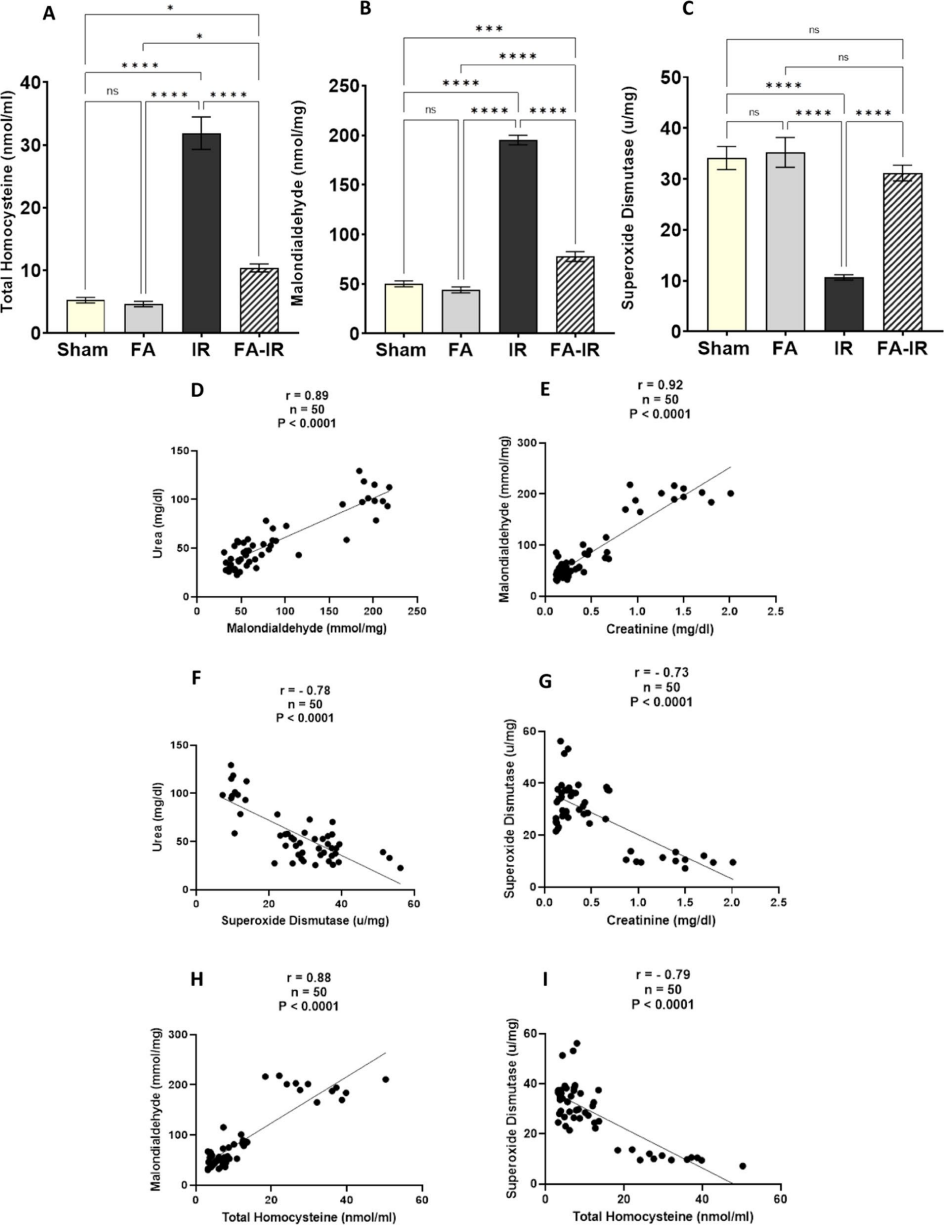
**A**–**C** Estimation of homocysteine and oxidative stress markers in the different studied groups. Plasma level of total Hcy (**A**), renal tissue level of MDA (**B**), and SOD (**C**). Values are expressed as the mean ± SEM. ns: nonsignificant, *p < 0.05, ****p < 0.0001. **D**–**I** Pearson’s correlation and simple linear regression of pooled values of all studied groups show a significant positive correlation between MDA and urea (**D**) and creatinine (**E**). Additionally, there was a significant negative correlation between SOD and urea (**F**) and creatinine (**G**). Significant positive correlation between Hcy and MDA (**H**). Significant negative correlation between Hcy and SOD (**I**). *n*: number of values = 50, r: Pearson’s correlation coefficient

Plasma total homocysteine levels were significantly elevated in the IR group compared to both the sham and FA groups (*p* < 0.0001 for both). In the FA-IR group, plasma homocysteine was significantly lower than that in the IR group (*p* < 0.0001) but remained significantly higher than that in both control groups (*p* < 0.05 for both). In the FA group, plasma homocysteine was insignificantly different compared to the sham group (Fig. 2A).

In Fig. 2B, the renal tissue MDA level showed a significant increase in the IR group compared to both the sham and FA groups (*p* < 0.0001 for both). In the FA-IR group, MDA was significantly decreased compared to that in the IR group (*p* < 0.0001), but it was significantly higher than that in both the sham and FA groups (*p* < 0.001 and < 0.0001, respectively). In the FA group, MDA showed insignificant changes when compared to the sham group. Renal tissue MDA showed significant positive correlations with serum urea and creatinine and plasma total homocysteine (Fig. 2D, E, H).

Renal tissue SOD activity levels showed a significant decline in the IR group compared to both the sham and FA groups (*p* < 0.0001 for both). In the FA-IR group, SOD activity was significantly higher than that in the IR group (*p* < 0.0001), but it was insignificant compared to both the sham and FA groups. Moreover, in the FA group, SOD activity was insignificant compared to that in the sham group (Fig. 2C). Renal tissue SOD activity showed significant negative correlations with serum urea and creatinine and plasma total homocysteine (Fig. 2F, G, I).

### Changes in apoptotic parameter levels in renal tissue

**Fig. 3:**
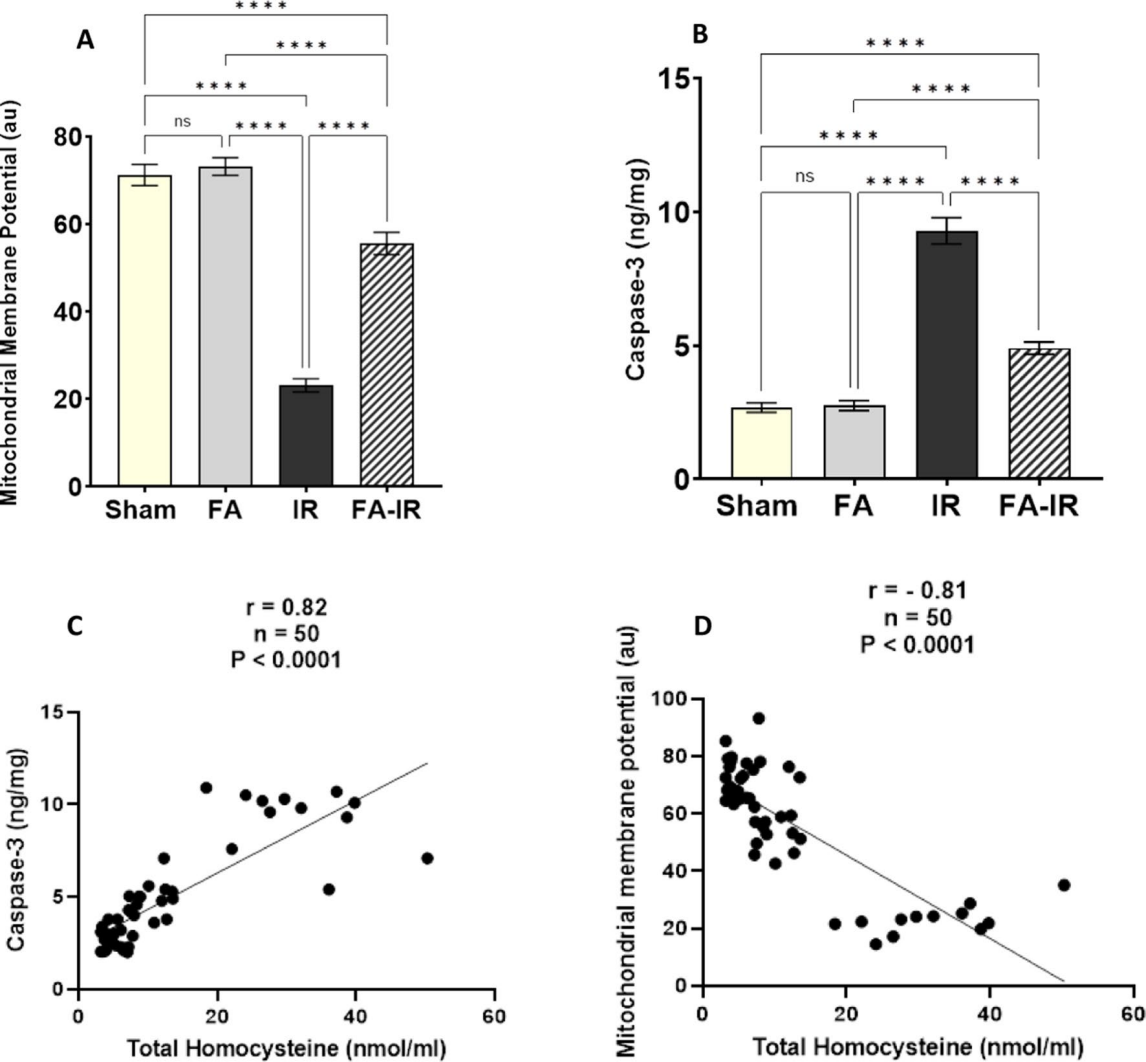
**A**, **B** Measurement of apoptotic and cell viability markers in the studied groups. MMP (**A**), and caspase-3 enzyme (**B**). Values are expressed as the mean ± SEM. ns: nonsignificant, ****p < 0.0001. **C**, **D** Pearson’s correlation and simple linear regression of pooled values of all studied groups show a significant positive correlation between Hcy and caspase-3 (**C**) and a significant negative correlation between Hcy and MMP (**D**). *n*: number of values = 50, r: Pearson’s correlation coefficient

Renal tissue MMP was significantly lower in the IR group than in both the sham and FA groups (*p* < 0.0001 for both). In the FA-IR group, MMP was significantly elevated compared to that in the IR group (*p* < 0.0001). On the other hand, it was significantly lower than that in both the sham and FA groups (*p* < 0.0001 for both). In the FA group, MMP showed insignificant differences when compared to the sham group (Fig. 3A). Renal tissue MMP levels showed a negative correlation with plasma total Hcy levels (Fig. 3D).

In Fig. 3B, the level of the apoptotic marker caspase-3 in renal tissue was significantly increased in the IR group compared to both the sham and FA groups (*p* < 0.0001 for both). In the FA-IR group, renal tissue caspase-3 was significantly decreased compared to that in the IR group (*p* < 0.0001). However, it remained significantly increased compared to both the sham and FA groups (*p* < 0.0001 for both). In the FA group, caspase-3 was statistically insignificant compared to the sham group. Renal tissue caspase 3 levels showed a positive correlation with plasma total Hcy levels (Fig. 3C).

### Changes in HMGB1 and NF-κB levels in renal tissue

**Fig. 4:**
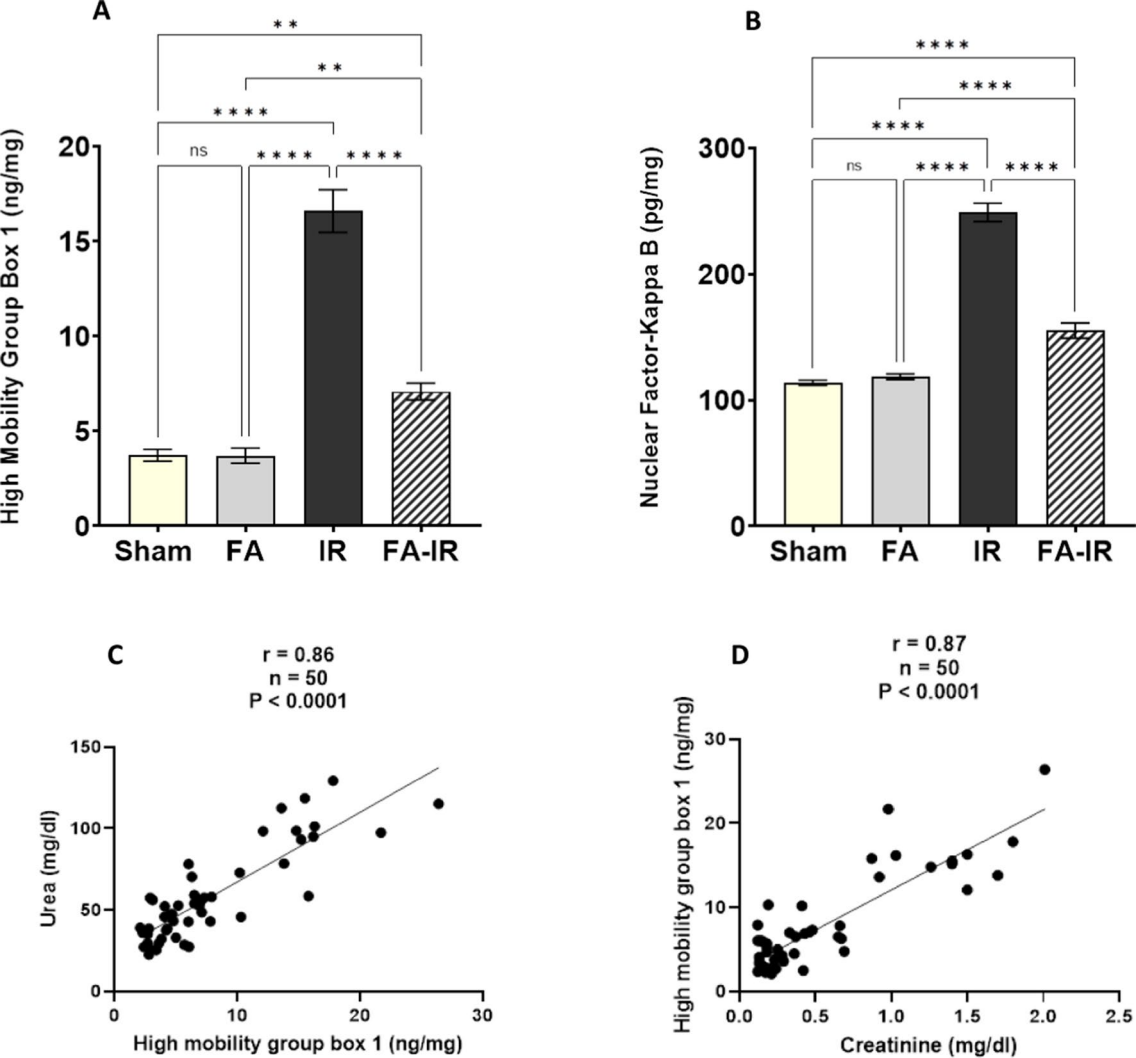
**A**, **B** Assessment of renal HMGB1 and downstream NF-κB under different conditions, HMGB1 (**A**) and NF-κB (**B**). Values are expressed as the mean ± SEM. ns: nonsignificant, **p < 0.01, ****p < 0.0001. **C**, **D** Pearson’s correlation and simple linear regression of pooled values of all different studied groups show a significant positive correlation between HMGB1 and urea (**C**). Significant positive correlation between HMGB1 and creatinine (**D**). *n*: number of values = 50, r: Pearson’s correlation coefficient

In Fig. 4A, renal tissue HMGB1 showed significant elevation in the IR group compared to both the sham and FA groups (*p* < 0.0001 for both). In the FA-IR group, HMGB1 levels were significantly lower than those in the IR group (*p* < 0.0001), but they were significantly higher than those in the sham and FA groups (*p* < 0.0001 for both). In the FA group, HMGB1 levels were not significantly different from those in the sham group. Renal tissue HMGB1 showed a significant positive correlation with serum urea (Fig. 4C) and serum creatinine levels (Fig. 4D).

Renal tissue NF-κB levels were significantly increased in the IR group compared to both the sham and FA groups (*p* < 0.0001 for both). In the FA-IR group, the NF-κB level was significantly decreased compared to that in the IR group (*p* < 0.0001). However, it was significantly higher in the FA-IR group than in both the sham and FA groups (*p* < 0.0001 for both). In the FA group, the NF-κB level showed nonsignificant changes compared to the sham group (Fig. 4B).

## Discussion

RIRI is a major cause of AKI that has been associated with a high risk of morbidity and mortality [22]*.* AKI triggers a series of biochemical and molecular changes that elicit inflammatory processes and apoptotic changes, leading to injurious effects in the kidneys as well as other organs [4]. The pathophysiological mechanisms underpinning RIRI are copious and intermingled together, e.g., oxidative stress, production of inflammatory mediators, and cell death [5]. Checking for a natural agent that could tackle RIRI at diverse molecular pathways with minimal or no side effects is very enticing. FA is a superb candidate that embraces all these criteria. In our study, FA conferred a protective effect on renal function through its antioxidative and antiapoptotic properties, which subsequently affected HMGB1 expression and its downstream inflammatory target molecule NF-kB.

The marked elevation in the classical renal function markers (i.e., serum creatinine and urea) observed in the IR group confirmed the success of our technique in the induction of renal injury. Meanwhile, both the sham group and FA-treated group expressed normal levels for both markers, implying preserved kidney function and underscoring the safe nature of the FA dose used on the kidney. Interestingly, FA treatment of the group subjected to IR injury rendered the kidney function markers significantly lower than those of the IR group by approximately 70%; however, they were still higher than those of the sham and FA-treated groups. This effect emphasizes the beneficial advantage of FA treatment, while its concurrent limits as a solo treatment in normalizing kidney function. The histopathological analysis for the IR group revealed extremely atrophic glomeruli associated with a loss of the tubular brush border, scattered totally necrotic proximal tubules with edematous epithelial lining, and marked intratubular hyaline casts. Moreover, clear signs of apoptosis were presented all over the cortico-medullary area. In contrast, FA treatment in the IR group could blunt these deleterious histological changes to maintain almost normal glomerular size and the tubular brush border, thus preserving most of the renal filtration and reabsorption function.

The dose chose according to previous experimental work [17], which is considered relatively higher than the normal daily intake (2.1 mg/kg) claimed by some research groups [23], but still much lower than the nephrotoxic doses used to induce kidney injury, which starts from 250 mg/kg (fivefold higher than the dose given in our experiment) [14]. Importantly, the safety nature of the dose used was reflected on the biochemical analysis for renal functions as well as the histological examination for the kidney from the folic acid only treated group. For human beings the RDA for folic acid is between 400 and 600 µg, even with high oral doses reaching 15 mg/day, there have been no substantiated reports of side effects [24]. High doses of folic acid can be used cautiously as a therapeutic dose for some clinical condition. Indeed, we do not recommend the usage of a high dose on human being, however, pushing the folic acid to its upper limits, which varies considerably between species, could impose beneficial effects in certain clinical conditions.

The role of oxidative stress in the induction of organ damage following ischemia‒reperfusion has been thoroughly described in the literature [25]. However, the full molecular mechanisms underlying oxidative stress damage still need to be explored. Homocysteine acts as a pro-oxidative agent that induces tissue damage by promoting local oxidative stress mediated by the presence of a thiol group that undergoes autoxidation rapidly whenever oxygen and metal ions are available, producing ROS. This oxidative activity becomes more accelerated by the activation of the NADPH oxidase enzyme, which leads to more ROS generation [26]*.* Interestingly, our results showed a sixfold increase in the serum level of Hcy in the rats subjected to IR compared to their control mates in both the sham and FA-only treated groups*.* A previous study documented a reduction in renal folate transporters following IR, which negatively affects the bioavailability of folic acid [27]*.* It is well known that folic acid is crucial in the biochemical reaction converting Hcy to methionine, thus helping to lower the plasma Hcy level [27]. The striking elevation in the serum level of Hcy following IR observed in our study could be a natural consequence of the aforementioned folate receptor reduction, thus reducing the folic acid serum concentration, which subsequently leads to Hcy trapping and prevents its further conversion to methionine. Meanwhile, Hcy was significantly lowered in the IR group treated with folic acid compared to the IR group, although it remained double that in the sham and FA only treated group.

To confirm the oxidative stress, we chose to assess another two markers: MDA, which is the end product of peroxidation of the cell membrane polyunsaturated fatty acids by ROS, and the antioxidant marker SOD. In the IR group, MDA was significantly higher than that in the control groups (almost fourfold), which confirms the involvement of lipid peroxidation and oxidative stress in IR pathology. The involvement of oxidative stress in the induction of kidney injury in our model was supported by the positive correlation between MDA levels and kidney function markers, i.e., creatinine and urea*.* Meanwhile, in the FA-IR group, MDA was lowered by 60% compared to the IR group but still higher (almost 1.5-fold) than the control groups. This could be attributed to the protective antioxidant effect of FA in this group [29] which reduced the abundance of ROS, thus protecting the kidneys from their deleterious effects [28]*.*

On the other hand, SOD levels in renal tissue were markedly reduced in the IR group by almost 65% compared with the other three groups. This low SOD could hinder renal safety measures and increase vulnerability to ROS-induced injury. This was demonstrated by the negative correlation between SOD and kidney function test levels. The reduction in SOD could be due to HHcy, which is accused of its downregulation in addition to other antioxidant enzymes, e.g., glutathione peroxidase, thus being involved in the pathophysiology of RIRI [6]. The negative correlation between Hcy levels and SOD supported this view*.* Meanwhile, SOD showed no significant difference between the sham-operated, FA-treated and FA-IR groups*.* These comparable levels reflect the high impact of FA at this dose and duration on alleviating SOD depletion and maintaining renal antioxidant capacity. Consistently, FA administration in a rat model of testicular IR was associated with a significant increase in the tissue level of SOD and a decrease in the tissue level of MDA. Therefore, FA administration to the FA-IR group could restore the balance between free radical generation and antioxidant defenses [29]*.*

The imbalance between the two arms determines the redox status in the organs, i.e., ROS generation, and antioxidative agents result in protein and DNA damage and trigger cellular apoptosis [27]. HHcy was reported to induce apoptosis via ROS generation and P38 mitogen-activated protein [32] By following this thread for deeper exploration of the IRI mechanism, we checked the level of caspase 3 in the renal tissue as well as MMP as a cellular activity and viability marker. Caspase 3 revealed a marked increase in the IR group compared to the other groups. FA treatment for the IR group could significantly lower the caspase 3 level; however, it remained higher than that of the control groups. Meanwhile, MMP was markedly reduced in the IR group. This reduction was lessened via FA treatment. These changes could be explained, partially, by the upstream negative influence of FA on Hcy levels or by acting directly on the mitochondria, reducing its permeability and decreasing the expression of proapoptotic proteins [33] This was also signified by the positive correlation between Hcy and caspase 3 and the negative correlation between MMP and Hcy.

As a next step, we investigated the influence of oxidative stress and the apoptotic state on HMGB1 and its downstream NF-kB molecule. HMGB1 is a nonhistone DNA chaperone protein that is aberrantly found extranuclear upon active secretion or passive release [10]. In agreement with other work [11]*,* our results confirmed the elevation of HMGB1 and NF-kB following renal IR. The uncertainty about its impact on kidney function was deciphered by the administration of neutralizing HMGB1 antibody in an ischemic-reperfusion AKI experimental model, which conferred an undoubtful renoprotective effect. HMGB1 has become a central molecule guiding many intermingled pathophysiological mechanisms leading to AKI [30]. HMGB1 was significantly lower in the FA-IR group than in the untreated group. This beneficial and novel influence of FA positively correlates with renal function*.* We speculate that the reduction in HMGB1 observed in the treated group was attributed to the antioxidative and antiapoptotic effects of FA [14]. Finally, once released from the cells, HMGB1 binds to cell surface receptors, inducing a reaction as a prototypical damage-associated molecular pattern (DAMP) [31]*. *Classic HMGB1 receptors include RAGE, TLRs (TLR2, TLR4, and TLR9), CXCR4, and T-cell immunoglobulin mucin-3 (TIM-3) leukocytes. Additionally, infiltrating leukocytes and cancer cells can secrete HMGB1 in response to hypoxia, injury, inflammatory stimuli, or environmental factors. This loop promotes inflammatory responses and the development of increasing inflammation [9]*.* The proinflammatory effect of the HMGB1-RAGE axis is significantly associated with the NF-κB pathway, which involves extracellular signal-regulated kinase 1 and 2 (ERK1/2) and p38 MAPK. Moreover, activated NF-κB translocates to the nucleus and interacts with DNA as a p65/p50 heterodimer, which enhances proinflammatory cytokine expression [32]**.** As a natural consequence of HMGB1 level reduction following FA treatment, NF-kB was markedly reduced compared to the untreated group, which can positively affect the trajectory of renal function [30], which again was confirmed in our study by the positive correlation found between HMGB1 and kidney function markers. HMGB1 is an intermediate key molecule in the pathophysiology of IR, residing between the oxidative stress induced by IR and inflammatory and apoptotic phases [33]*.*

## In conclusion

Our study revealed a novel beneficial protective mechanism of FA following renal IR exerted by reducing HMGB1 and subsequently inducing inflammation secondary to its Hcy-lowering effect and both antioxidative and antiapoptotic properties. This renoprotective effect employed by multiple mechanisms rendered the use of FA a very tempting approach to ameliorate renal injury following IR.

### Supplementary Information

**Additional file 1: Table S1.** Histopathological scores of renal changes in 10 sections examined from different studied groups. In each group, we reported the number of sections showing each score. **Table S2. **composition of the standard rat chow diet used in our study. **Figure S1. (A-D):** Photomicrographs of renal cortex of different studied groups (Masson’s Trichrome x400): Sham group: showing few collagen fibers between glomerular capillaries (*), surrounding renal corpuscle (↑) and between the renal tubules (▲) (A). FA group: showing few collagen fibers between glomerular capillaries (*), surrounding renal corpuscle (↑) and between the renal tubules (▲) (B). IR group: showing increased collagen fibers between glomerular capillaries (*), surrounding renal corpuscle (↑) and in between renal tubules (▲). Increased collagen deposition in the lumen of some tubules (*) (C). FA-IR group: showing a mild increase in collagen fibers between glomerular capillaries (*), few collagen fibers surrounding the renal corpuscle (↑) and between renal tubules (▲) (D). Scale bar is 50 µm. **Figure S2. (A-D):** Photomicrographs of the renal cortex of different studied groups (PAS x 400). Sham group: PAS-positive brush border of cells lining PCTs (↑), the basement membrane of renal tubules (blue arrow) and the parietal layer of the Bowman capsule (red arrow)(A). FA group: PAS-positive brush border of cells lining PCTs (↑), the basement membrane of renal tubules (blue arrow) and the parietal layer of the Bowman capsule (red arrow) (B). IR group: showing loss of brush border of cells of PCTs (↑), the basement membrane of the tubules (blue arrow) and the parietal layer of Bowman capsule (red arrow) showing increased positive PAS reaction (C). FA-IR group: showing focally interrupted PAS-positive brush border in most of the PCTs (↑), PAS-positive basement membrane of the tubules (blue arrow) and parietal layer of Bowman capsule (red arrow) (D). Scale bar is 50 µm.

## Data Availability

The authors confirm that the data supporting the findings of this study are available within the article and its supplementary materials.

## Abbreviations

AKI: Acute kidney injury
CKD: Chronic kidney disease
DAMPs: Damage-associated molecular patterns
DNA: Deoxyribonucleic acid
ELISA: Enzyme-linked immunosorbent assay
ERK1/2: Extracellular signal-regulated kinase 1 and 2
FA: Folic acid
GSH: Glutathione
H&E: Hematoxylin and Eosin stain
H_2_O_2_: Hydrogen peroxide
Hcy: Homocysteine
HHcy: Hyper-homo-cysteinemia
HMGB1: High mobility group box 1
I/R, IR: Ischemia/reperfusion
MAPK: Mitogen-activated protein kinases
MASRI: Faculty of Medicine Ain Shams University Research Institute
MDA: Malondialdehyde
MMP: Mitochondrial Membrane Potential
NADPH: Nicotinamide adenine dinucleotide phosphate
NF-Κb: Nuclear factor-kappa B
PAS: Periodic Acid Schiff
PCT: Proximal convoluted tubules
RAGE: Receptors for advanced glycation end-products
RIRI: Renal ischemia–reperfusion injury
ROS: Reactive oxygen species
SEM: Standard error of mean
SOD: Superoxide dismutase
TIM-3: T-cell immunoglobulin mucin-3
TLRs: Toll-like receptors
