# Supplementary material for: Impact of folic acid supplementation on ischemia‒reperfusion-induced kidney injury in rats: folic acid prophylactic role revisited
Source: J Physiol Sci. 2024 Feb 7;74:7. doi: 10.1186/s12576-024-00900-z (PMC10848562; doi:10.1186/s12576-024-00900-z)
Supplement: Supplementary file 1 — Additional file 1: Table S1. Histopathological scores of renal changes in 10 sections examined from different studied groups. In each group, we reported the number of sections showing each score. Table S2. composition of the standard rat chow diet used in our study. Figure S1. (A-D): Photomicrographs of renal cortex of different studied groups (Masson’s Trichrome x400): Sham group: showing few collagen fibers between glomerular capillaries (*), surrounding renal corpuscle (↑) and between the renal tubules (▲) (A). FA group: showing few collagen fibers between glomerular capillaries (*), surrounding renal corpuscle (↑) and between the renal tubules (▲) (B). IR group: showing increased collagen fibers between glomerular capillaries (*), surrounding renal corpuscle (↑) and in between renal tubules (▲). Increased collagen deposition in the lumen of some tubules (*) (C). FA-IR group: showing a mild increase in collagen fibers between glomerular capillaries (*), few collagen fibers surrounding the renal corpuscle (↑) and between renal tubules (▲) (D). Scale bar is 50 µm. Figure S2. (A-D): Photomicrographs of the renal cortex of different studied groups (PAS x 400). Sham group: PAS-positive brush border of cells lining PCTs (↑), the basement membrane of renal tubules (blue arrow) and the parietal layer of the Bowman capsule (red arrow)(A). FA group: PAS-positive brush border of cells lining PCTs (↑), the basement membrane of renal tubules (blue arrow) and the parietal layer of the Bowman capsule (red arrow) (B). IR group: showing loss of brush border of cells of PCTs (↑), the basement membrane of the tubules (blue arrow) and the parietal layer of Bowman capsule (red arrow) showing increased positive PAS reaction (C). FA-IR group: showing focally interrupted PAS-positive brush border in most of the PCTs (↑), PAS-positive basement membrane of the tubules (blue arrow) and parietal layer of Bowman capsule (red arrow) (D). Scale bar is 50 µm. [file 12576_2024_900_MOESM1_ESM.pptx]

## Slide 1
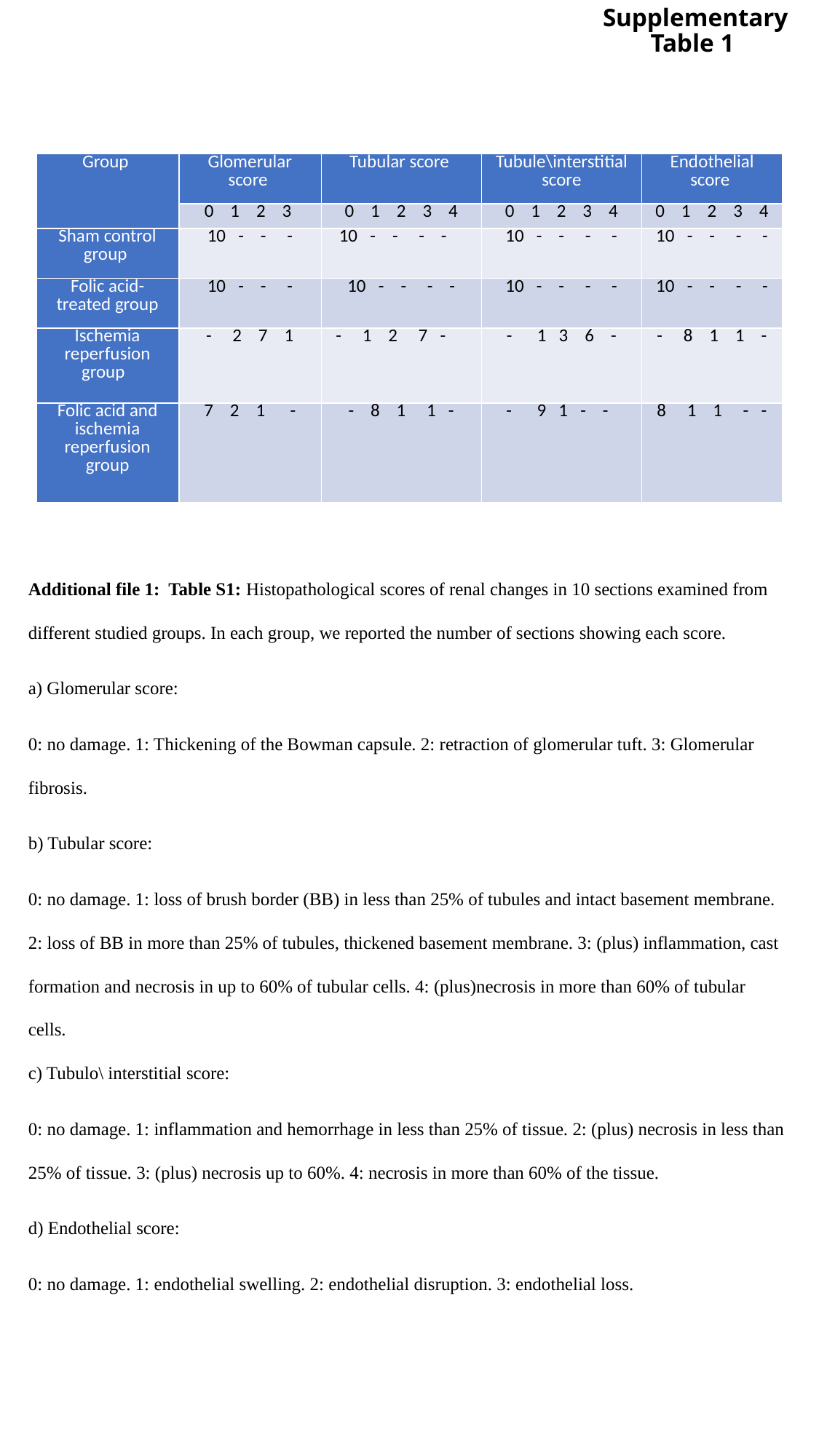

Supplementary Table 1
| Group | Glomerular score | Tubular score | Tubule\interstitial score | Endothelial score |
| --- | --- | --- | --- | --- |
| | 0 1 2 3 | 0 1 2 3 4 | 0 1 2 3 4 | 0 1 2 3 4 |
| Sham control group | 10 - - - | 10 - - - - | 10 - - - - | 10 - - - - |
| Folic acid-treated group | 10 - - - | 10 - - - - | 10 - - - - | 10 - - - - |
| Ischemia reperfusion group | - 2 7 1 | - 1 2 7 - | - 1 3 6 - | - 8 1 1 - |
| Folic acid and ischemia reperfusion group | 7 2 1 - | - 8 1 1 - | - 9 1 - - | 8 1 1 - - |
Additional file 1: Table S1: Histopathological scores of renal changes in 10 sections examined from different studied groups. In each group, we reported the number of sections showing each score.
a) Glomerular score:
0: no damage. 1: Thickening of the Bowman capsule. 2: retraction of glomerular tuft. 3: Glomerular fibrosis.
b) Tubular score:
0: no damage. 1: loss of brush border (BB) in less than 25% of tubules and intact basement membrane. 2: loss of BB in more than 25% of tubules, thickened basement membrane. 3: (plus) inflammation, cast formation and necrosis in up to 60% of tubular cells. 4: (plus)necrosis in more than 60% of tubular cells.
c) Tubulo\ interstitial score:
0: no damage. 1: inflammation and hemorrhage in less than 25% of tissue. 2: (plus) necrosis in less than 25% of tissue. 3: (plus) necrosis up to 60%. 4: necrosis in more than 60% of the tissue.
d) Endothelial score:
0: no damage. 1: endothelial swelling. 2: endothelial disruption. 3: endothelial loss.

## Slide 2
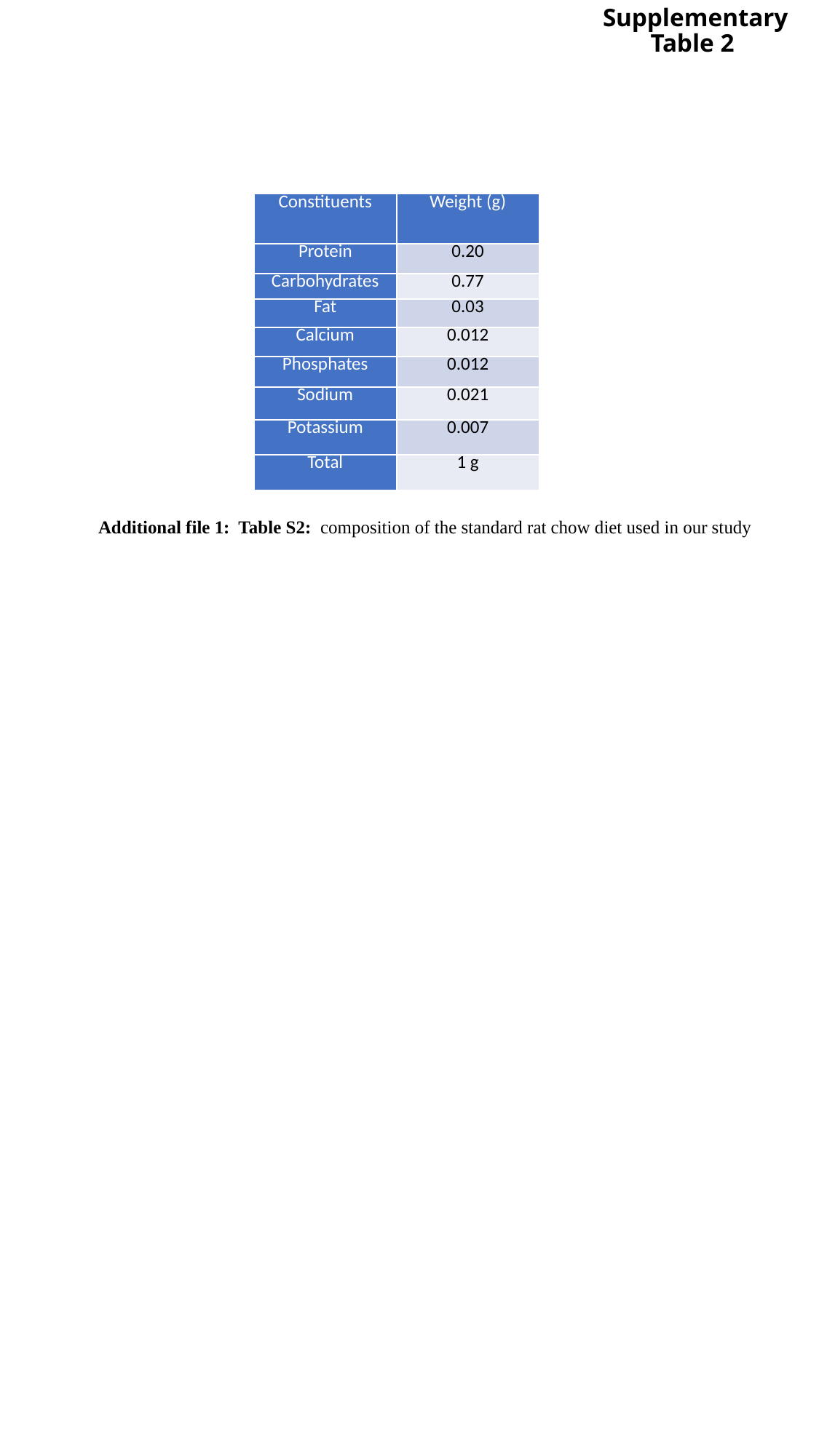

Supplementary Table 2
| Constituents | Weight (g) |
| --- | --- |
| Protein | 0.20 |
| Carbohydrates | 0.77 |
| Fat | 0.03 |
| Calcium | 0.012 |
| Phosphates | 0.012 |
| Sodium | 0.021 |
| Potassium | 0.007 |
| Total | 1 g |
Additional file 1: Table S2: composition of the standard rat chow diet used in our study

## Slide 3
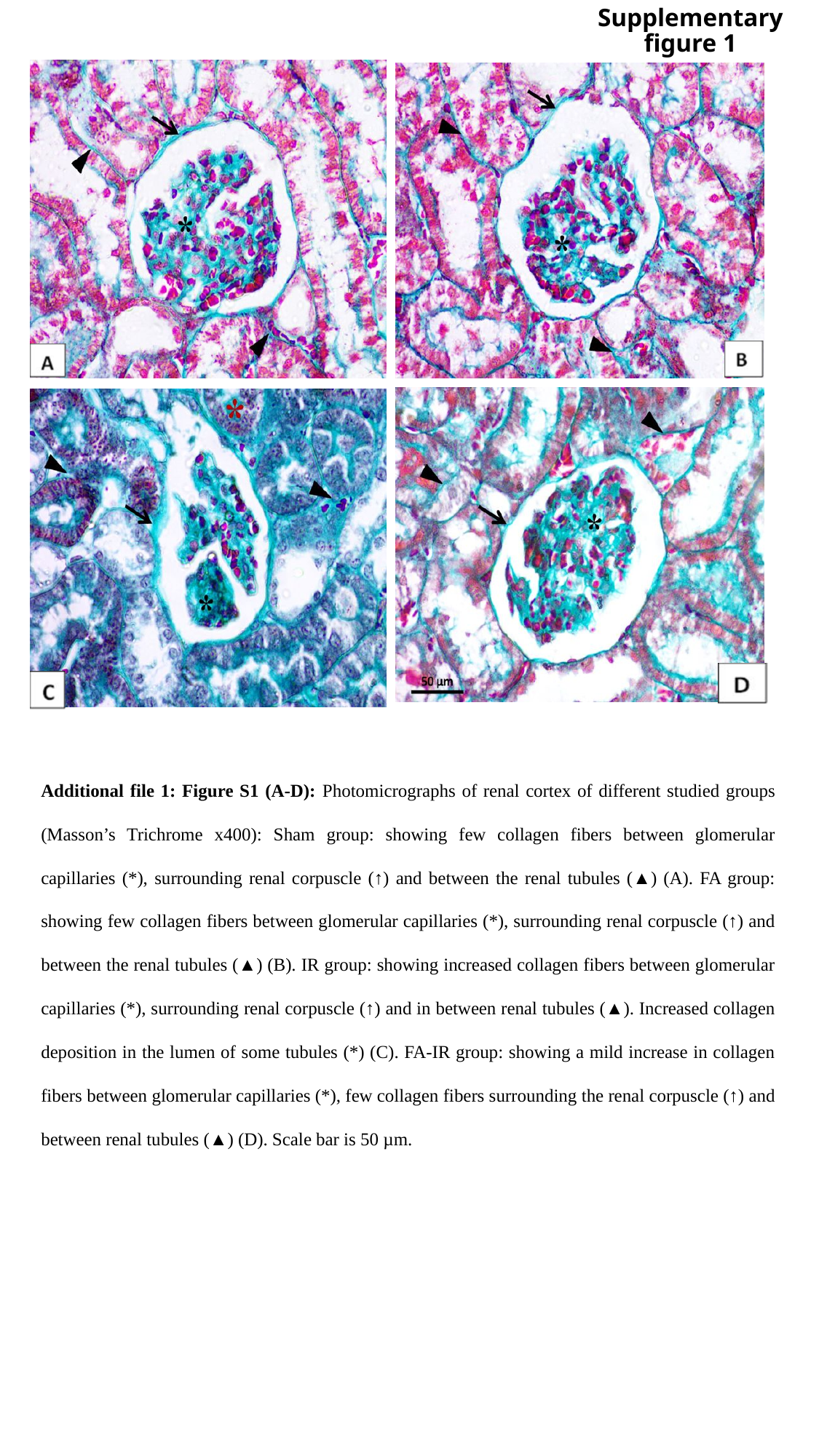

Supplementary figure 1
Additional file 1: Figure S1 (A-D): Photomicrographs of renal cortex of different studied groups (Masson’s Trichrome x400): Sham group: showing few collagen fibers between glomerular capillaries (*), surrounding renal corpuscle (↑) and between the renal tubules (▲) (A). FA group: showing few collagen fibers between glomerular capillaries (*), surrounding renal corpuscle (↑) and between the renal tubules (▲) (B). IR group: showing increased collagen fibers between glomerular capillaries (*), surrounding renal corpuscle (↑) and in between renal tubules (▲). Increased collagen deposition in the lumen of some tubules (*) (C). FA-IR group: showing a mild increase in collagen fibers between glomerular capillaries (*), few collagen fibers surrounding the renal corpuscle (↑) and between renal tubules (▲) (D). Scale bar is 50 µm.

## Slide 4
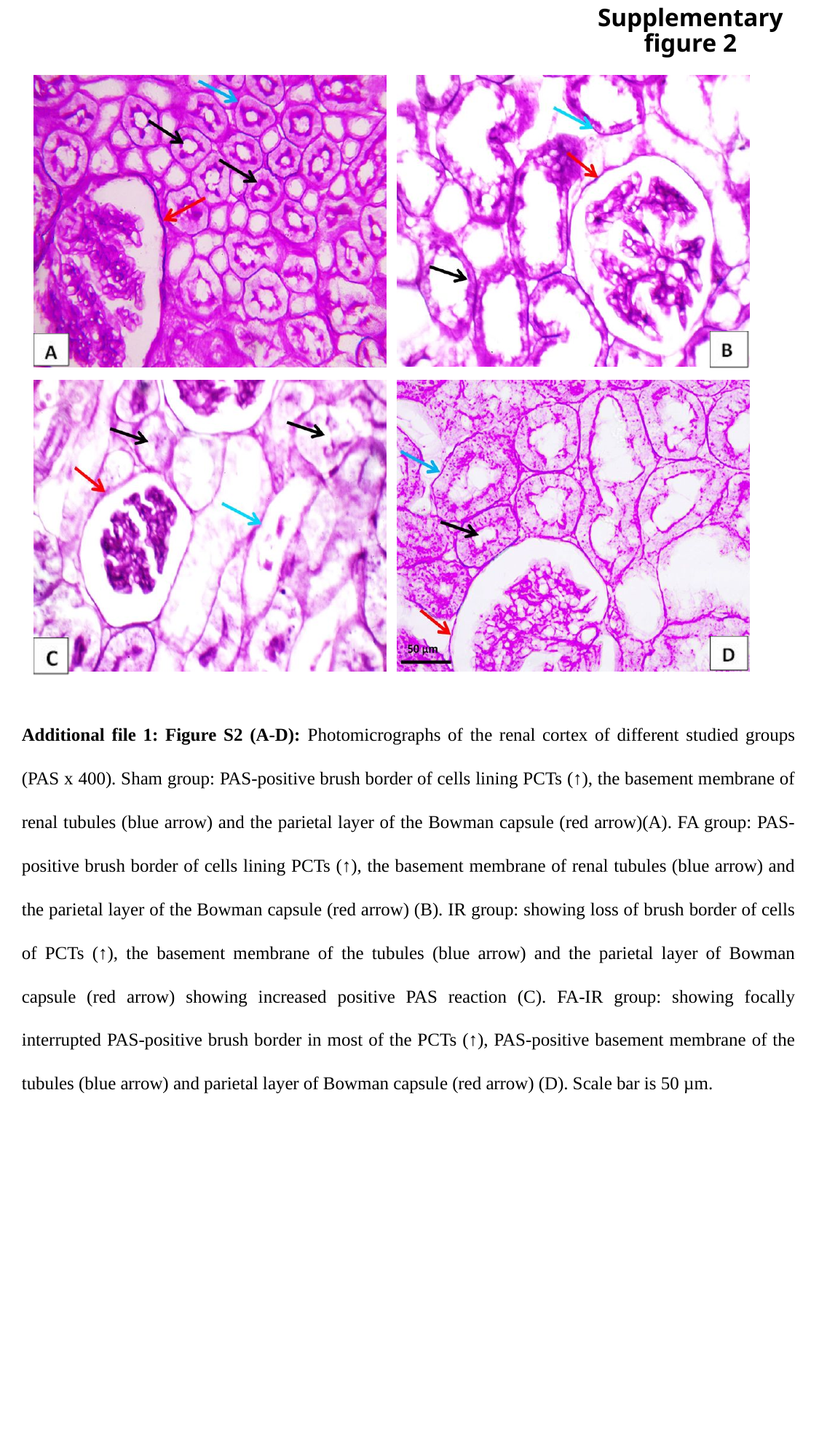

Supplementary figure 2
Additional file 1: Figure S2 (A-D): Photomicrographs of the renal cortex of different studied groups (PAS x 400). Sham group: PAS-positive brush border of cells lining PCTs (↑), the basement membrane of renal tubules (blue arrow) and the parietal layer of the Bowman capsule (red arrow)(A). FA group: PAS-positive brush border of cells lining PCTs (↑), the basement membrane of renal tubules (blue arrow) and the parietal layer of the Bowman capsule (red arrow) (B). IR group: showing loss of brush border of cells of PCTs (↑), the basement membrane of the tubules (blue arrow) and the parietal layer of Bowman capsule (red arrow) showing increased positive PAS reaction (C). FA-IR group: showing focally interrupted PAS-positive brush border in most of the PCTs (↑), PAS-positive basement membrane of the tubules (blue arrow) and parietal layer of Bowman capsule (red arrow) (D). Scale bar is 50 µm.
